# PDE2 Is a Novel Target for Attenuating Tumor Formation in a Mouse Model of UVB-Induced Skin Carcinogenesis

**DOI:** 10.1371/journal.pone.0109862

**Published:** 2014-10-16

**Authors:** Jamie J. Bernard, You-Rong Lou, Qing-Yun Peng, Tao Li, Yao-Ping Lu

**Affiliations:** Susan Lehman Cullman Laboratory for Cancer Research, Department of Chemical Biology, Ernest Mario School of Pharmacy, Rutgers, The State University of New Jersey, Piscataway, New Jersey, United States of America; University of Tennessee, United States of America

## Abstract

Our previous studies demonstrated that the topical application of caffeine is a potent inhibitor of UVB-induced carcinogenesis and selectively increases apoptosis in tumors but not in non-tumor areas of the epidermis in mice that are at a high risk for developing skin cancer. While this effect is mainly through a p53 independent pathway, the mechanism by which caffeine inhibits skin tumor formation has not been fully elucidated. Since caffeine is a non-specific phosphodiesterase inhibitor, we investigated the effects of several PDE inhibitors on the formation of sunburn cells in mouse skin after an acute exposure to ultraviolet light B (UVB). The topical application of a PDE2 inhibitor, erythro-9-(2-hydroxy-3-nonyl) adenine hydrochloride (EHNA hydrochloride), stimulated epidermal apoptosis compared to control (*P*<0.01) and to a greater extent than caffeine whereas a PDE4 inhibitor attenuated the epidermal apoptosis compared to control (*P*<0.01). Since PDE2 hydrolyzes cyclic nucleotides, mainly cGMP, the effects of EHNA hydrochloride on epidermal apoptosis following UVB exposure may be mediated, in part, by increased cGMP signaling. Data demonstrated that the topical application of dibutyryl cGMP stimulated epidermal apoptosis (*P*<0.01) following an acute exposure to UVB. Treating UVB-pretreated mice topically with 3.1 µmole or 0.8 µmole of EHNA hydrochloride attenuated tumor formation to a greater extent than treating with 6.2 µmole caffeine when these compounds were applied once a day, five days a week for 18 weeks. These observations suggest a novel role for PDE2 in UVB-induced tumorigenesis and that PDE2 inhibitors that mediate cGMP signaling may be useful for the prevention and treatment of skin cancer.

## Introduction

Sunlight-induced non-melanoma skin cancer is the most common cancer in the United States [1]. Wavelengths in the ultraviolet B light (UVB) range (290–320 nm) induce erythema, burns and are primarily responsible for these cancers. Although most skin cancers are squamous cell carcinomas and basal cell carcinomas that are easily cured if detected early, many people still die from these cancers, as well as from the more dangerous sunlight-induced melanomas. The development of strategies to prevent or cure UVB-induced cancers would have a major impact in decreasing the total load of human cancer.

Our previous studies demonstrated that the topical application of caffeine is a potent inhibitor of UVB-induced carcinogenesis by selectively increasing apoptosis in tumors but not in non-tumor areas of the epidermis in mice that are at a high risk for developing skin cancer [2]–[5]. While this effect is mainly through a p53 independent pathway [3], the mechanism by which caffeine inhibits skin tumor formation has not been fully elucidated.

Caffeine (1,3,7-trimethylxanthine) is a broad spectrum, non-selective phosphodiesterase (PDE) inhibitor that is metabolized into dimethylxanthines. Naturally occurring methylxanthines were the first inhibitors of cyclic nucleotide PDEs to be discovered. Caffeine acts by competing with adenine, the purine base of DNA, for access to the catalytic site of PDEs and prevents PDEs from inactivating cAMP or cGMP. PDEs hydrolyze cyclic nucleotides by breaking the diester bond that connects the 5′ carbon to the 3′ carbon of the ribose. Eleven families of PDE isoenzymes can be distinguished that differ in their biochemical properties, their localization and their affinities for cAMP, cGMP or both [6]–[8].

cAMP and cGMP are intracellular second messengers that modulate several signaling pathways and control several functions in cells. A novel approach to anti-tumor therapy is to modulate cAMP and cGMP with PDE inhibitors as cAMP and cGMP are negative regulators of cell growth and aberrant signaling has been shown to play an important role in various carcinomas and hematological malignancies [7], [9]. Since caffeine is a non-selective PDE inhibitor, we hypothesized that caffeine inhibits UVB-induced skin cancer by inhibiting PDEs to modulate the intracellular cyclic nucleotides cAMP and/or cGMP.

The present study used several pharmacologically selective and non-selective PDE inhibitors to assess the role of PDE inhibition on epidermal apoptosis following an acute exposure to UVB. The presence of apoptotic epidermal cells (apoptotic sunburn cells) indicated the potential for anti-cancer effects of a compound *in vivo* [2], [10]–[14]. Comparing structurally similar PDE inhibitors to caffeine, we found differential effects on the percentage of UVB-induced apoptotic sunburn cells. The selective PDE2 inhibitor, EHNA hydrochloride [15], significantly increased the percentage of UVB-induced apoptotic sunburn cells compared with caffeine and control while the selective PDE4 inhibitor, ICI 63,197 [16], significantly decreased the percentage. PDEs hydrolyze cyclic nucleotides and, due differences in affinities, PDE2 inhibitors, specifically EHNA hydrochloride, have been shown to mainly increase cGMP signaling whereas PDE4 inhibitors have been shown to increase cAMP signaling. Despite the differences observed with EHNA hydrochloride and ICI 63,197, the topical application of exogenous dibutyryl cGMP or dibutyryl cAMP derivatives both increased epidermal apoptosis after an acute exposure to UVB. EHNA hydrochloride was then tested for its efficacy in attenuating UVB-induced skin carcinogenesis. Results demonstrated that EHNA hydrochloride potently inhibited UVB-induced skin tumor formation.

## Materials and Methods

### Animals

These studies were approved by the Rutgers IACUC. CO_2_ and cervical dislocation were used as a method of euthanasia. The protocol approval number is 88-056. Female SKH-1 hairless mice (6–7 weeks old) were purchased from Charles River Breeding Laboratories and kept in our animal facility for 1 week before use. Mice were maintained on a 12 h light/12 h dark cycle and provided food (Laboratory Chow 5001 from the Ralston Purina company) and water *ad libitum.*

### Exposure to UVB

The UV lamps used (FS72T12-UVB-HO; National Biological Corp., Twinsburg, Ohio) emitted UVB (280–320 nm; 75–80% of total energy) and UVA (320–375 nm; 20–25% of total energy). The dose of UVB was quantified with a UVB Spectra 305 dosimeter (Daavlin Co., Byran, OH). The radiation was further calibrated with a model IL-1700 research radiometer/photometer (International Light Inc., Newburyport, MA). For PDE apoptosis studies, mice were exposed to a single dose of 30 mJ/cm^2^ of UVB and then treated topically with PDE inhibitors. For the PDE carcinogenesis studies, mice were treated with UVB (30 mJ/cm^2^) twice a week for 20 weeks and UVB exposure was stopped. Following UVB, mice were treated topically with PDE inhibitors. More experimental details on treatments can be found in the two sections below.

### PDE apoptosis studies

Female SKH-1 hairless mice were exposed to a single dose of 30 mJ/cm^2^ of UVB. Immediately following UVB and at 30 and 120 minutes post-UVB exposure, mice were treated topically with MMPX, EHNA hydrochloride, Cilostamide, ICI 63,197, T0156, zaprinast, dipyridamole or caffeine in 100 µl acetone:water (9∶1). The animals were killed at 6 hrs after UVB exposure (peak time point for UVB-induced apoptosis). Apoptotic sunburn cells in the epidermis were determined morphologically by cell shrinkage and nuclear condensation as we have performed previously. PDE inhibitors were purchased from Tocris Bioscience (Ellisville, MO).

### PDE carcinogenesis studies

Female SKH-1 hairless mice (7 to 8 weeks old, 30 per group) were treated with UVB (30 mJ/cm^2^) twice a week for 20 weeks and UVB exposure was stopped. These high risk mice were then treated topically with 3.1 µmole or 0.8 µmole of erythro-9-(2-hydroxy-3-nonyl) adenine hydrochloride (EHNA hydrochloride) or 6.2 µmole caffeine in 100 µl acetone:water (8∶2) once a day five days a week for 18 weeks. These mice were killed at 28 weeks after last dose of UVB. Body weight and skin tumor formation (percent mice with tumors, number of tumors per mouse and tumor volume per mouse) were measured every 2 weeks. The counting of all tumors and measurement of tumor size were performed blind with respect to treatment group. Tumor volume was determined by measuring the three-dimensional size (height, length, and width) of each mass. The average of the three measurements was used as the diameter. The radius (r) was determined, and the tumor volume was calculated by: volume = 4πr^3^/3.

### Statistical analysis

The Student’s *t*-test was used for simple comparisons of two groups. The analysis of variance (ANOVA) model was used for comparisons of multiple treatment groups with a common control group using Microsoft Excel. For all statistical tests, a *P* value of <0.05 was accepted as statistical significance. All data are means +/− SEM.

## Results

### A PDE2 inhibitor stimulates and a PDE4 inhibitor attenuates epidermal apoptosis after an acute exposure to UVB

**Figure 1:**
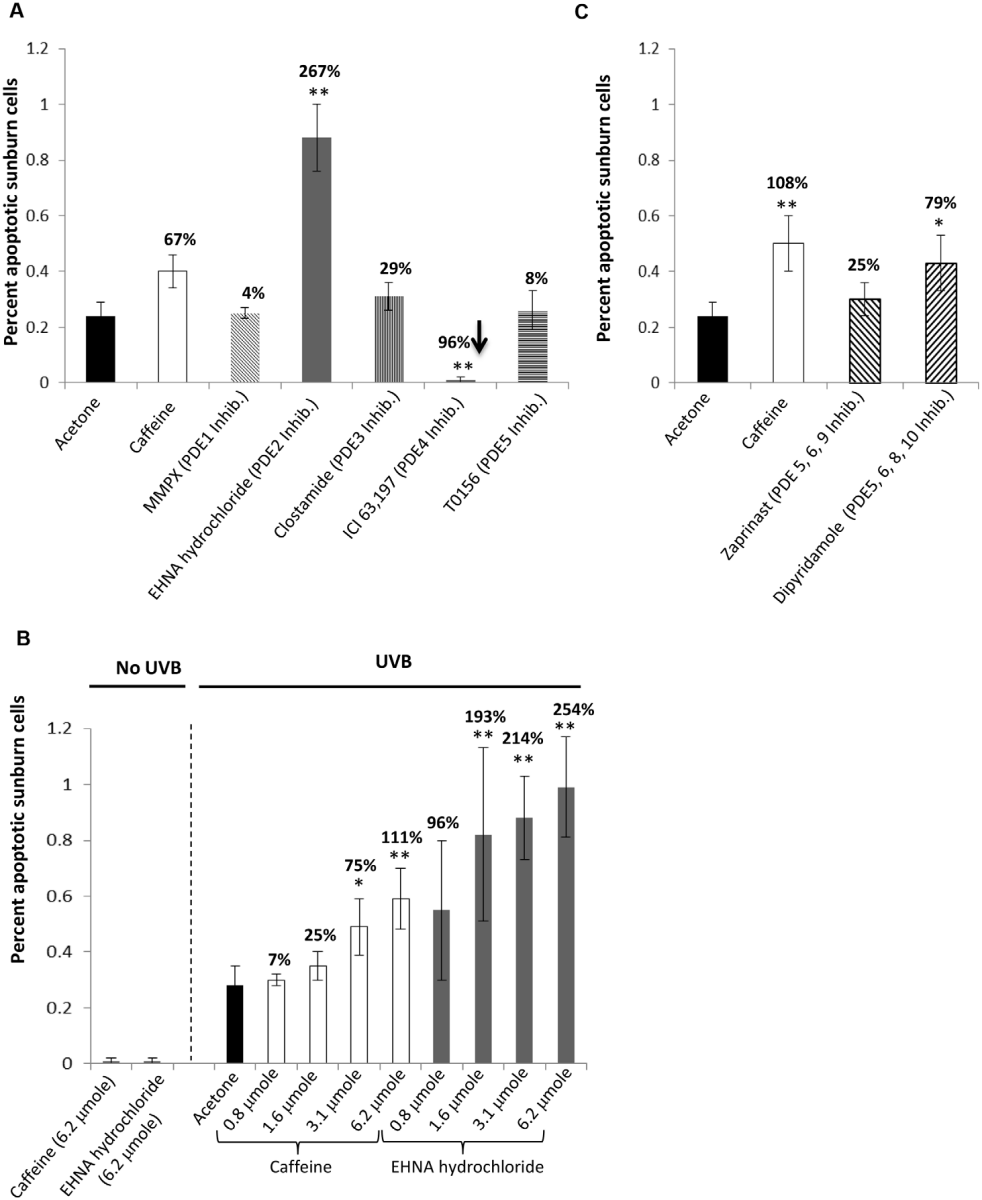
Effects of phosphodiesterase inhibitors on epidermal apoptosis after an acute exposure to UVB. **A.** Female SKH-1 hairless mice (7 to 8 weeks old, 5 per group) were treated topically with caffeine or different PDE inhibitors at a concentration of 3.1 µmole (in 100 µl acetone:water (9∶1) right after a single dose of 30 mJ/cm^2^ of UVB and at 30 and 120 min later. The animals were killed at 6 hrs after UVB. Apoptotic sunburn cells in the epidermis were determined morphologically. Value is percent increase compared with acetone control except for the value on ICI 63,197 which is percent decrease compared with acetone control (**P<0.01). All data are mean ± SD. **B.** Mice were treated as described in **A**, but 6.2 µmole of PDE inhibitors were used instead of 3.1 µmole. Value is percent increase compared with acetone control (*P<0.05, **P<0.01). All data are mean ± SD. **C.** Mice were treated as described in **A**, but different doses of caffeine and EHNA hydrochloride were used. Value on EHNA hydrochloride bars is percent increase compared with caffeine (*P<0.05, **P<0.01). All data are mean ± SD. N.S. is not significant.

Previous studies determined that caffeine, a non-specific phosphodiesterase (PDE) inhibitor, attenuated UVB-induced carcinogenesis [4] therefore, we tested the effect of several different selective and non-selective PDE inhibitors on epidermal apoptosis following an acute exposure to UVB. The presence of apoptotic epidermal cells (apoptotic sunburn cells) was determined to be an indicator for the anti-cancer effects of a compound *in vivo* [2], [10]–[14]. Immediately following UVB and at 30 and 120 minutes post-UVB exposure, female SKH-1 mice were treated topically with MMPX, erythro-9-(2-hydroxy-3-nonyl) adenine hydrochloride (EHNA hydrochloride), Cilostamide, ICI 63,197, T0156, zaprinast, dipyridamole or caffeine in 100 µl acetone:water (9∶1) at a concentration of 3.1 µmole (Fig. 1A) or 6.2 µmole (Fig. 1B). The animals were then killed at 6 hrs following UVB exposure (*peak* time point for *UVB*-induced *apoptosis*). Apoptotic sunburn cells in the epidermis were determined morphologically by cell shrinkage and nuclear condensation. The results showed that a selective cGMP-activated PDE2 inhibitor, EHNA hydrochloride had a more pronounced stimulatory effect than caffeine on UVB-induced apoptosis (Fig. 1A). Topical application of 3.1 µmole EHNA enhanced UVB-induced apoptosis by 267% (*P*<0.01), whereas topical application of same amount of caffeine (3.1 µmole) only enhanced apoptosis by 68% (*P*<0.01) compared with the acetone control group. Topical application of 3.1 µmole of EHNA hydrochloride induced 0.01% apoptotic sunburn cells in non-UVB irradiated mouse epidermis. The significant increase in apoptotic sunburn cells in EHNA hydrochloride-treated epidermis was validated with a dose-response experiment, where several doses of EHNA hydrochloride were compared to the same doses of caffeine. Except at the lowest dose (0.8 µmole), EHNA hydrochloride significantly stimulated UVB-induced apoptosis when compared to caffeine (Fig. 1C). EHNA hydrochloride at 0.8, 1.6, 3.1, and 6.2 µmole stimulated UVB-induced apoptosis 83, 134, 80, and 68% more than the same dose of caffeine (Fig. 1C).

Dipyridimole, a PDE 5, 6, 8, 10, 11 inhibitor, also stimulated epidermal apoptosis 79% more than the acetone control (*P*<0.05) although not to the same extent as the same dose of caffeine (6.2 µmole) (Fig. 1B). Conversely, topical application of a selective cGMP-insensitive, cAMP-mediated PDE4 inhibitor, 2-amino-6-methyl-4-propyl-[1], [2], [4]triazolo[1,5-a]pyrimidin-5(4H)-one (ICI 63,197), almost completely inhibited UVB-induced apoptosis (96% inhibition) when compared with the acetone control group (*P*<0.01, Fig. 1A). These data demonstrate that UVB-induced apoptosis is dependent which PDEs are inhibited.

### Effects of phosphodiesterase inhibitors and cyclic nucleotides on epidermal apoptosis after an acute exposure to UVB

**Figure 2:**
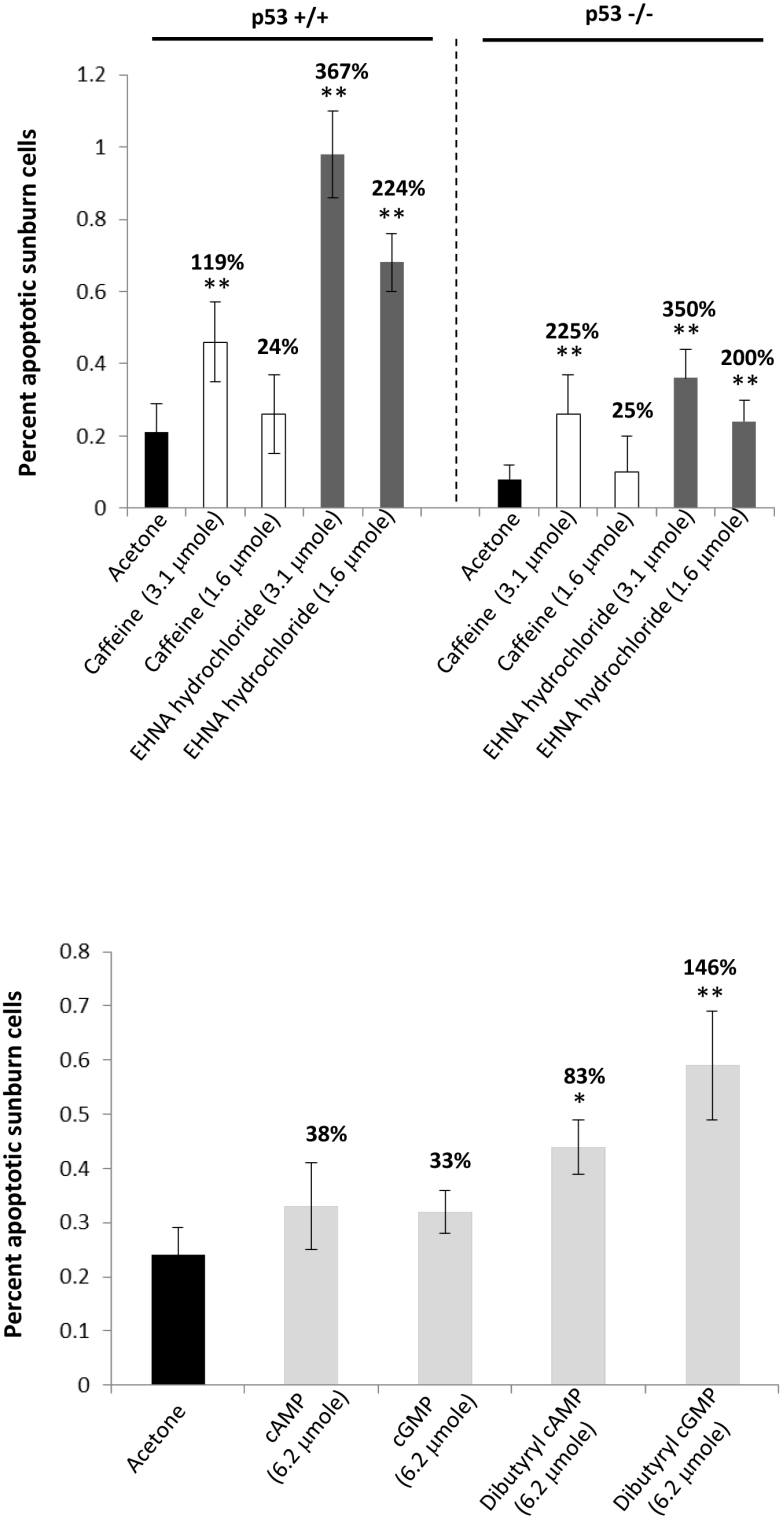
Effects of phosphodiesterase inhibitors and cyclic nucleotides on epidermal apoptosis after an acute exposure to UVB. **A.** Female p53 wild-type or p53 knockout SKH-1 hairless mice (7 to 8 weeks old, 5 per group) were treated topically with caffeine or different PDE inhibitors (in 100 µl acetone:water (9∶1) right after a single dose of 30 mJ/cm^2^ of UVB and at 30 and 120 min later. The animals were killed at 6 hrs after UVB. Apoptotic sunburn cells in the epidermis were determined morphologically. Value is percent increase compared with acetone control (**P<0.01). In the p53 wild-type mice only, EHNA hydrochloride significantly induces apoptotic sunburn cells compared with caffeine (^$^ *P*<0.05). All data are mean ± SD. **B.** Female SKH-1 mice (8 to 9 weeks old, 5 per group) were treated topically with 100 µl of acetone:water (9∶1) or 6.2 µmole of compounds in 100 µl of acetone:water (9∶1) immediately after 30 mJ/cm^2^ of UVB irradiation and at 30 and 120 min later. The animals were killed 6 hours later. Apoptotic sunburn cells in the epidermis were determined morphologically. Value is percent increase compared with acetone control (*P<0.05, **P<0.01).

To mimic a more physiologically relevant model of skin cancer, we repeated this study utilizing congenic p53 knockout (−/−) hairless mice since most UVB-induced skin tumors are characterized by p53 mutations. p53 wild-type (+/+) littermates were used as a control. The dose of caffeine and EHNA hydrochloride was reduced to 1.6 and 3.1 µmole as the previous experiment indicated that EHNA hydrochloride was still able to significantly stimulate epidermal apoptosis at these doses (Fig. 2A). Topical application of EHNA hydrochloride dose-dependently induced apoptotic sunburn cells in the UVB-irradiated mouse epidermis in p53 (+/+) (224 and 367%) and p53 (−/−) (200 and 350%) mice similar to that which was observed in the SKH-1 mice (Fig. 2A). Interestingly, EHNA hydrochloride significantly stimulated apoptotic sunburn cells compared with caffeine in the p53 (+/+) mice (^$^ *P*<0.05) but not in the p53 (−/−) mice indicating that the number of epidermal apoptotic cells may have plateaued in the p53 (−/−) which are more sensitive to UVB (unpublished observation). These data suggest that a PDE2 inhibitor will attenuate tumor formation independently of p53 expression.

Since the PDE2 inhibitor EHNA hydrochloride, which can enhance cGMP expression, induced epidermal apoptosis after an acute exposure to UVB, we hypothesized that the topical application of cGMP would also induce apoptotic sunburn cells. To test this hypothesis, immediately following UVB and at 30 and 120 minutes post-UVB exposure, female SKH-1 mice were treated topically with either cAMP or cGMP at 6.2 µmole to mimic the dose of caffeine that stimulated epidermal apoptosis. cAMP or cGMP had no significant effect on epidermal apoptosis (Fig. 2B) and therefore, dibutyryl cAMP or dibutyryl cGMP (6.2 µmole) were applied. These are membrane permeable analogs of cAMP and cGMP that are used experimentally to mimic the intracellular actions of cAMP and cGMP. Dibutyryl cGMP increased the percentage of apoptotic sunburn cells by 146%, similar to that of EHNA hydrochloride (*P*<0.01) (Fig. 2B). Interestingly, dibutyryl cAMP also increased the percentage of apoptotic sunburn cells (86%), but less significantly than dibutyryl cGMP (*P*<0.05) suggesting that intracellular cGMP is the critical mediator of the apoptotic sunburn response induced by EHNA hydrochloride (Fig. 2B). These results suggest that the cGMP-activating PDE2 inhibitor, EHNA hydrochloride, and caffeine may inhibit UVB-induced skin carcinogenesis by regulating the levels of cGMP.

### A PDE2 selective inhibitor attenuates UVB-induced carcinogenesis

**Figure 3:**
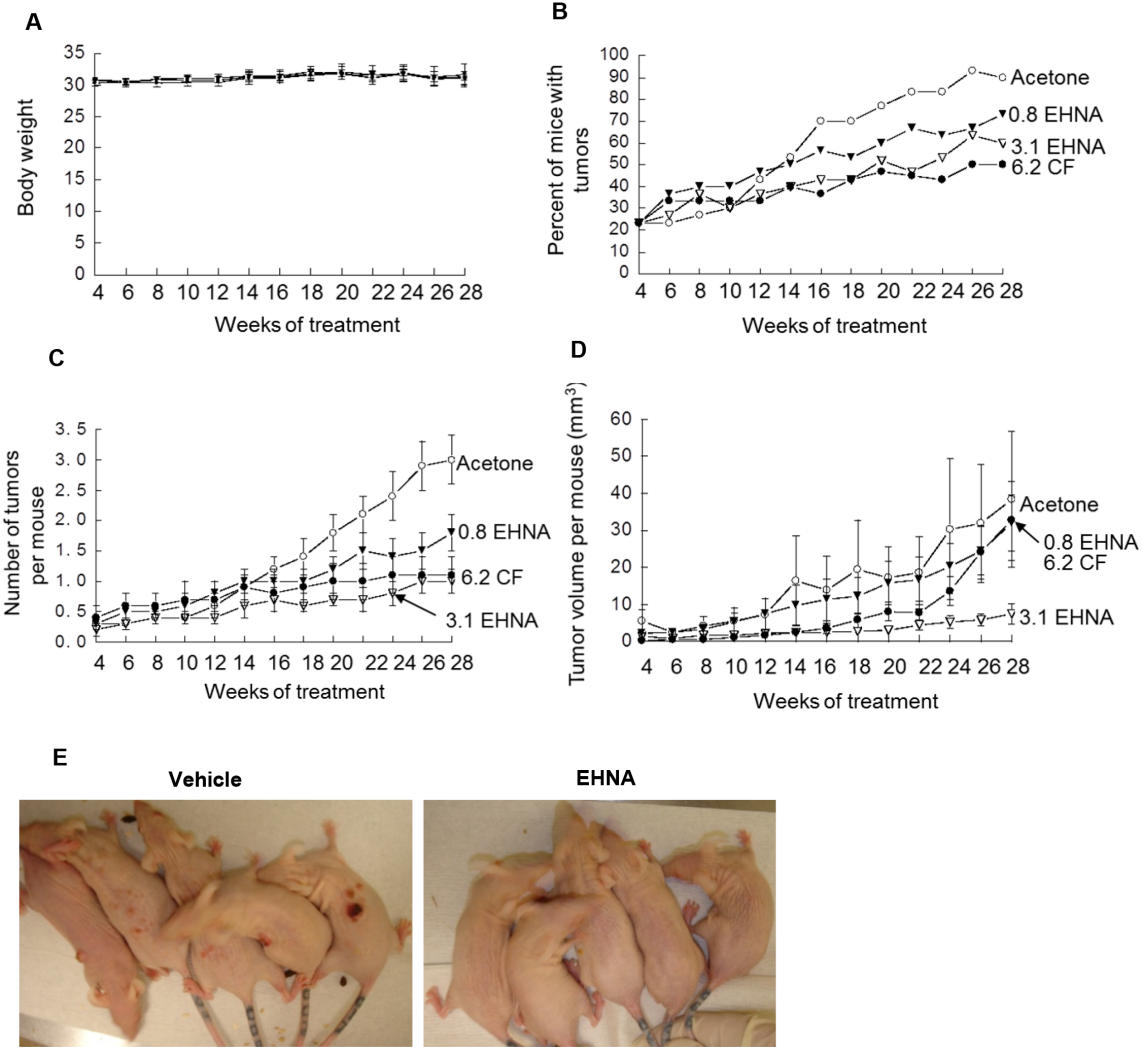
Comparing the effects of caffeine (CF) and EHNA hydrochloride (EHNA) on tumor formation. SKH-1 hairless mice (7 to 8 weeks old, 30 per group) were treated with UVB (30 mJ/cm^2^) twice a week for 20 weeks and UVB exposure was stopped. These high risk mice were then treated topically with 3.1 µmole or 0.8 µmole of erythro-9-(2-hydroxy-3-nonyl) adenine hydrochloride (EHNA) or 6.2 µmole caffeine (CF) in 100 µl acetone:water (8∶2) once a day five days a week for 18 weeks. These mice were killed at 28 weeks after last dose of UVB. The number and size of tumors in each animal was measured each 2 weeks for 24 weeks. **A.** Graph shows body weight analysis. Treatments did not influence body weight. **B.** Graph shows the percent of mice with tumors. Mice show an increase in the percent of tumors over time. **C.** Graph shows the number of tumors per mouse. The results indicate that EHNA hydrochloride treatment significantly reduced tumor number per mouse compared with acetone treatment at 28 weeks (*P*<0.05 for the 0.8 µmole dose and *P*<0.01 for the 3.1 µmole dose). **D.** Graph shows the tumor volume (mm^3^) per mouse. The results indicate that EHNA hydrochloride treatment significantly reduced tumor volume per mouse compared with acetone treatment at 28 weeks (*P*<0.01). **E.** Picture shows tumor formation on the dorsal skin of SKH-1 mice that were treated with either vehicle or EHNA hydrochloride (EHNA, 3.1 µmole).

Since the apoptotic studies indicated that the cGMP-activating PDE2 inhibitor, EHNA hydrochloride, induced more epidermal apoptosis when compared to caffeine, a non-specific PDE inhibitor we next determined the effect of different doses of EHNA hydrochloride on UVB-induced carcinogenesis. Female SKH-1 hairless mice were treated with UVB (30 mJ/cm^2^) twice a week for 20 weeks and UVB exposure was stopped. These high risk mice were then treated topically with 3.1 µmole or 0.8 µmole of EHNA hydrochloride in 100 µl acetone:water (8∶2) once a day five days a week for 18 weeks. Acetone was used as the vehicle control and 6.2 µmole caffeine was used as a positive control. These mice were killed at 28 weeks after their last dose of UVB. The number and size of tumors in each animal was measured every 2 weeks for 24 weeks. The results indicated that acetone or EHNA hydrochloride had no effect on body weight in UVB-initiated animals, indicting little to no toxicity (Fig. 3A) however, EHNA hydrochloride-treated mice had an increased latency for the development of tumors compared with acetone-treated mice (Fig. 3B). After treatment for 6 weeks, the tumor incidence in the EHNA hydrochloride-treated mice was always lower than that in the acetone-treated mice (Fig. 3A). In both the acetone control and EHNA hydrochloride groups, the number of tumors per mouse increased with time (Fig. 3B), but after 6 weeks of treatment, EHNA hydrochloride-treated mice kept a low level of tumors per mouse while tumors quickly increased in the acetone-treated mice. These results indicated that EHNA hydrochloride treatment significantly reduced tumor number per mouse compared with acetone treatment at 28 weeks (*P*<0.05 for the 0.8 µmole dose and *P*<0.01 for the 3.1 µmole dose). In addition, the tumor volume per mouse increased quickly in the EHNA hydrochloride-treated mice compared with the acetone-treated mice (Fig. 3C). These results indicated that EHNA hydrochloride treatment significantly reduced tumor volume per mouse compared with acetone treatment at 28 weeks (*P*<0.01).

### EHNA hydrochloride attenuates both malignant and non-malignant tumor formation to a greater extent than caffeine

Histological examination of tumors demonstrated that caffeine-treatment and EHNA-hydrochloride-treatment yielded 46% and 63% less mice with non-malignant tumors (squamous cell papillomas and keratoacanthomas) than acetone-treatment and 43% and 67% less mice with malignant tumors (squamous cell carcinomas) at the 6.2 µmole dose (Table 1). Even at the lower dose of EHNA hydrochloride (3.1 µmole), 19% less mice had non-malignant tumors and 43% less mice had malignant tumors than in the acetone control group. Caffeine-treated mice and EHNA-hydrochloride-treated mice had 67% and 76% less non-malignant tumors per mouse and 20% and 72% less malignant tumors per mouse compared to acetone-treated mice at the 6.2 µmole dose. EHNA-hydrochloride-treated mice (3.1 µmole) had 30% less non-malignant tumors and 52% less malignant tumors per mouse. EHNA hydrochloride reduced tumor volume per mouse (non-malignant and malignant) by 79% and 94% at the low and high doses whereas caffeine decreased tumor volume by only 29% (Table 2). Overall, EHNA hydrochloride at both the low and high doses reduced the number of malignant tumors and total tumor volume per mouse to a greater extent than caffeine. The difference between skin tumors in the caffeine-treated and EHNA hydrochloride-treated groups can be observed (Fig. 3E).

**Table 1:** Effect of topical applications of EHNA on the incidence and multiplicity of histologically characterized skin tumors in high risk SKH-1 mice previously treated with UVB.

		Squamous cellpapillomas		Keratoacanthomas		Total nonmalignant tumors		Squamous cell carcinomas		Total tumors	
Treatment	No. ofmice	% micewithtumors	Tumorsper mouse	% micewithtumors	Tumorsper mouse	% micewithtumors	Tumors permouse	% micewithtumors	Tumorspermouse	% micewithtumors	Tumorspermouse
Acetone	28	0	0	89	3.32±0.66	89	3.32±0.66	21	0.25±0.10	89	3.57±0.69
CF	25	4.0 (0)	0.04±0.04 (0)	48 (46)	1.08±0.35 (67)	48 (46)	1.12±0.36 (66)	12 (43)	0.20±0.13 (20)	48 (46)	1.32±0.42 (63)
EHNA (3.1 µmole)	27	3.7 (0)	0.04±0.04 (0)	30 (66)	0.81±0.31 (76)	33 (63)	0.85±0.31 (74)	7 (67)	0.07±0.05 (72)	33 (63)	0.93±0.32 (74)
EHNA (0.8 µmole)	25	4.0 (0)	0.04±0.04 (0)	72 (19)	2.28±0.48 (31)	72 (19)	2.32±0.48 (30)	12 (43)	0.12±0.07 (52)	72 (19)	2.44±0.49 (32)

Female SKH-1 mice (7–8 weeks old) were irradiated with UVB (30 mJ/cm^2^) twice weekly for 20 weeks, and UVB irradiation was stopped. These tumor-free mice with a high risk of developing skin tumors were treated topically with 3.1 µmole or 0.8 µmole of erythro-9-(2-hydroxy-3-nonyl) adenine hydrochloride (EHNA hydrochloride) or 6.2 µmole caffeine in 100 µl acetone:water (8∶2) once a day five days a week for 16 weeks. These mice were killed at 36 weeks after the last dose of UVB and all tumors were characterized by histopathology and the size of each tumor was determined. Each value is the mean ± SE, and the numbers in parentheses represent percent decrease.

**Table 2:** Effect of topical applications of EHNA on the size of tumors in high risk SKH-1 mice previously treated with UVB.

		Squamous cellpapillomas	Keratoa-canthomas	Total non-malignanttumors	Squamous cellcarcinomas	Total tumors
Treatment	No. ofmice	Tumor volume permouse (mm^3^)	Tumor volume permouse (mm^3^)	Tumor volumeper mouse (mm^3^)	Tumor volume permouse (mm^3^)	Tumor volumeper mouse (mm^3^)
Acetone	28	0±0	5.9±1.9	5.9±1.9	23.1±12.1	28.9±13.1
CF	25	0.2±0.2 (0)	3.3±1.6 (44)	3.3±1.5 (44)	17.1±11.8 (26)	20.6±11.7 (29)
EHNA (3.1 µmole)	27	0.0±0.0 (0)	0.7±0.3 (88)	0.7±0.3 (88)	0.9±0.7 (96)	1.7±0.8 (94)
EHNA (0.8 µmole)	25	0.0±0.0 (0)	3.8±1.1 (36)	3.8±1.1 (36)	2.2±1.3 (90)	6.0±1.7 (79)

Female SKH-1 mice (7–8 weeks old) were irradiated with UVB (30 mJ/cm^2^) twice weekly for 20 weeks, and UVB irradiation was stopped. These tumor-free mice with a high risk of developing skin tumors were treated topically with 3.1 µmole or 0.8 µmole of erythro-9-(2-hydroxy-3-nonyl) adenine hydrochloride (EHNA hydrochloride) or 6.2 µmole caffeine in 100 µl acetone:water (8∶2) once a day five days a week for 16 weeks. These mice were killed at 36 weeks after the last dose of UVB and all tumors were characterized by histopathology and the size of each tumor was determined. Each value is the mean ± SE, and the numbers in parentheses represent percent increase.

## Discussion

Skin is barrier between the environment and internal organs important for the preservation of body homeostasis through a highly regulated cutaneous neuroendocrine system [17]. Skin cancer is a growing health problem with more than two million new cases diagnosed annually [1]. The use and development of PDE inhibitors that modulate intracellular cyclic nucleotide signaling is a new therapeutic strategy for chemoprevention and chemotherapy [7], [18]–[20] however, the effects of PDE inhibitors on epidermal apoptosis and skin tumorigenesis have never been reported. This manuscript demonstrates that the PDE2 selective inhibitor, EHNA hydrochloride, induced epidermal apoptosis and attenuated UVB-induced carcinogenesis in a “high risk” mouse model of skin carcinogenesis. In this model, SKH-1 mice were exposed to UVB twice a week for 20 weeks, had no tumors, but were at a high risk of developing tumors during the next several months as previously described [4], [21]. The “high risk mouse” is a useful animal model that may be comparable to humans previously exposed to moderate/high levels of sunlight who have a high risk of developing skin cancers later in life even in the absence of continued heavy sunlight exposure. EHNA hydrochloride induced no toxicity and was efficacious at attenuating tumor formation at a 0.8 µmole dose. Moreover, this compound was able to stimulate epidermal apoptosis in the absence of functional p53, a characteristic of most skin tumors.

We began these studies to investigate the mechanism by which caffeine reduced tumor formation in our model of UVB-induced skin carcinogenesis. Caffeine is a non-specific PDE inhibitor. The first products of caffeine metabolism are all dimethylxanthines. Xanthines inhibit cyclic nucleotide PDEs that hydrolytically inactivate cAMP and cGMP. Inhibiting PDEs increases cAMP or cGMP to trigger a signal transduction cascade that activates cyclic nucleotide-dependent protein kinases A and G, and regulates ion channels and levels of scaffolding proteins. The aberrant regulation of cAMP and cGMP are common during carcinogenesis and the expression of these cyclic nucleotides have differential effects on cell growth and survival depending on the stage of cancer and the type of cell/tissue being studied [7], [9]. The 11 PDE isozyme families identified display unique substrate specificities and are classified by their preference for cAMP, cGMP or both which makes the development of selective PDE inhibitors an attractive means to prevent or treat cancer.

The huge commercial success of the PDE5 inhibitor, Viagra, has stimulated research and development of PDE -based therapies. PDE inhibitors are now studied for their use in several pathologies such pulmonary disease (COPD, asthma), sexual dysfunction, vascular disease, neurogenerative disease, depression, diabetes, rheumatoid arthritis and cancer [22]. ICI 63,197 (Rolipram), used clinically as an antidepressant compound, was shown to be a potent cAMP-PDE4 inhibitor [23]. ICI 63,197 promotes axonal regeneration and functional recovery [24], stimulates osteoclast formation [25], and activates mitochondrial apoptosis in chronic lymphocytic leukemia cells [26]. Studies have used EHNA hydrochloride as a tool to examine the role of PDE4 for calcium control in cardiac myocytes [27] and to reverse hypoxic pulmonary vasoconstriction in a perfused rat lung model [28]. The structure of EHNA hydrochloride, ICI 63,197 and caffeine are all very similar however, this is the first manuscript to test the use of ICI 63,197 and EHNA hydrochloride compared to caffeine in a model of carcinogenesis.

Our observations that the PDE4 inhibitor, ICI 63,197, which stimulates cAMP signaling, attenuated UVB-induced apoptosis while the PDE2 inhibitor, EHNA hydrochloride, which stimulates cGMP signaling, enhanced UVB-induced apoptosis and attenuated tumor formation in a mouse model of UVB-induced carcinogenesis are partly consistent with what has been reported for intracellular cyclic nucleotides in other types of epithelial cancers. For example, colorectal cancer cells have decreased basal levels of cGMP [29] and lung, bladder, ovarian, prostate, breast and colon cancers overexpress the transporter which exports cGMP from cells [30], [31]. This suggests that lower levels of cGMP are associated with epithelial cancers. In fact, several studies have demonstrated that cGMP signaling in epithelial cells stimulates apoptosis and inhibits proliferation to promote tumorigenesis [32]–[34]. In addition, cGMP signaling activates PKG which can then regulate other downstream targets that also have anti-proliferative and pro-apoptotic effects [35]–[37]. Conversely, cAMP signaling has been associated with oncogenic activity in epithelial tissues [38], [39].

In our study, topically applied dibutyryl cAMP or dibutyryl cGMP to mouse skin following UVB exposure increased the percentage of apoptotic sunburn cells. However, dibutyryl cGMP increased the percentage of epidermal apoptotic cells (146%) to a greater extent than dibutryl cAMP (83%). Based on our results with the PDE4 inhibitor, ICI 63,197, we expected dibutyryl cAMP to decrease the percentage of epidermal apoptotic cells. However, the topical application of exogenous intracellular cyclic nucleotide second messengers may have differential effects on epidermal apoptosis compared to that of endogenous intracellular cyclic nucleotide second messengers stimulated by PDE inhibitors. While the classical role of cGMP in skin is for vasodilation, the importance of cGMP and the differential roles of cAMP and cGMP signaling in skin cancer needs further exploration. Moreover, there is the potential for non-specific effects of EHNA hydrochloride and therefore future studies are planned to determine the effect of PDE2 knockdown or other PDE2 selective inhibitors such as IC 933 and BAY 60–7550 on skin cell transformation and tumor formation.

Herein, we demonstrated that PDE4 inhibition and PDE2 inhibition have opposing effects on the percentage of sunburn cells following an acute exposure to UVB. A PDE4 inhibitor attenuated apoptosis and a PDE2 inhibitor enhanced apoptosis. While caffeine is a broad-spectrum PDE inhibitor, it was not as potent as EHNA hydrochloride, a PDE2 inhibitor at attenuating UVB-induced tumor formation. This may be due to the ability of caffeine to inhibit not only PDE2, but also PDE4. Overall, these data suggest that PDE2 may be a novel target for inhibition of human skin tumors after years of sun exposure.
